# Development and Validation of an Immune-Related Signature for the Prediction of Recurrence Risk of Patients With Laryngeal Cancer

**DOI:** 10.3389/fonc.2021.683915

**Published:** 2021-12-16

**Authors:** Hang Zhang, Xudong Zhao, Jin Wang, Wenyue Ji

**Affiliations:** Department of Otolaryngology Head and Neck Surgery, Shengjing Hospital of China Medical University, Shenyang, China

**Keywords:** laryngeal cancer, recurrence, immune, signature, prognosis

## Abstract

**Objective:**

Our purpose was to develop and verify an immune-related signature for predicting recurrence risk of patients with laryngeal cancer.

**Methods:**

RNA-seq data of 51 recurrence and 81 non-recurrence laryngeal cancer samples were downloaded from TCGA database, as the training set. Microarray data of 34 recurrence and 75 non-recurrence cancer samples were obtained from GEO dataset, as the validation set. Single factor cox regression was utilized to screen prognosis-related immune genes. After LASSO regression analysis, an immune-related signature was constructed. Recurrence free survival (RFS) between high- and low- recurrence risk patients was presented, followed by ROC. We also evaluated the correlation between immune infiltration and the signature using the CIBERSORT algorithm. The genes in the signature were validated in laryngeal cancer tissues by western blot or RT-qPCR. After RCN1 knockdown, migration and invasion of laryngeal cancer cells were investigated.

**Results:**

Totally, 43 prognosis-related immune genes were identified for laryngeal cancer. Among them, eight genes were used for constructing a prognostic signature. High risk group exhibited a higher recurrence risk than low risk group. The AUC for 1-year was separately 0.803 and 0.715 in the training and verification sets, suggesting its well efficacy for predicting the recurrence. Furthermore, this signature was closely related to distinct immune cell infiltration. RCN1, DNAJA2, LASP1 and IBSP were up-regulated in laryngeal cancer. RCN1 knockdown restrained migrated and invasive abilities of laryngeal cancer cells.

**Conclusion:**

Our findings identify a reliable immune-related signature that can predict the recurrence risk of patients with laryngeal cancer.

## Introduction

Laryngeal cancer is a commonly diagnosed head and neck malignancy globally (1). It is induced by various risk factors, such as smoking, drinking and human papillomavirus infection (1). At present, the main treatment strategies for laryngeal cancer are surgery, radiotherapy as well as chemotherapy (2). Under the goals of maintaining speech and swallowing functions, some patients have experienced relapse and require salvage treatment. Recurrence is the main manifestation of treatment failure for laryngeal cancer (3). The prognosis of patients with recurrent laryngeal cancer is very poor. Even if salvage whole laryngectomy or re-radiotherapy is taken, patients who relapse within 2 years after initial treatment may still die from recurrence (3). Some clinical indicators have been reported as prognostic predictors for patients with recurrent head and neck carcinoma such as presence or absence of complications as well as tumor cell differentiation (4). Thus, how to reduce mortality and risk of recurrence remains a major clinical challenge for head and neck surgeons (5). Hence, it is urgent to probe into novel detection indicators that can predict patients’ recurrence.

Tumor, node, metastasis (TNM) stage is the main clinical tool to predict the risk of recurrence (6). However, in clinical practice, its predictive power is limited. Patients in the same stage often have different clinical outcomes due to the heterogeneous clinical pathological characteristics and tumor biology (7). Studies have reported that the TNM staging system cannot accurately evaluate the prognosis of patients with laryngeal cancer (7). In other tumor types, it also shows that including more clinical prognostic factors for prognostic analysis can make the results more accurate and reliable. The immune system plays a vital role in controlling tumor growth and progression. Infiltrating immune cells are highly heterogeneous in laryngeal cancer (8). The abnormal interaction between tumor cells and immune cells in the tumor microenvironment may promote the occurrence of tumors. For example, high density of tumor infiltrating lymphocytes is associated with a better prognosis of laryngeal carcinoma (9). With the development of molecular biology, prognostic models have been developed to predict the prognosis of laryngeal cancer. For example, Zhang et al. constructed a 4-lncRNA prognostic signature for laryngeal cancer (10). Ana Gabriela Jover-Esplá et al. developed a scoring system for predicting laryngeal cancer patients’ 5-year mortality (11). Nevertheless, there is still a lack of reliable molecular models for predicting the recurrence of patients with laryngeal cancer. Thus, in this study, we developed a reliable model to predict the risk of recurrence for patients with laryngeal cancer.

## Materials and Methods

### Data Collection and Pre-Processing

The level 3 RNA-seq data and the corresponding clinical information of 132 cases of laryngeal cancer (including 51 recurrence samples and 81 non-recurrence samples) were obtained from The Cancer Genome Atlas (TCGA; https://cancergenome.nih.gov). Microarray expression profiling of 109 laryngeal cancer patients was download from the Gene Expression Omnibus (GEO; accession: GSE27020) (12) *via* the GEOquery package, which was then annotated by the corresponding package from the bioconductor. This dataset was based on the platform of GPL96 [HG-U133A] Affymetrix Human Genome U133A Array. Among them, 34 patients relapsed after surgery. Only patients diagnosed with laryngeal cancer were included in our study. Those without complete clinical data (recurrence endpoint and recurrence time) were excluded. Data from different platforms were normalized by the limma package (13). TCGA cohort was the training set and GEO cohort was the validation set. **Table 1** lists the clinical characteristics of laryngeal cancer in the two cohorts.

**Table 1:** Clinical characteristics of laryngeal cancer in the two datasets.

Characteristics		GSE27020 (n = 109)	TCGA (n = 132)
Age (year)	≤60	43	62
	>60	66	70
Grade	1	44	6
	2	49	18
	3	16	30
	4a	0	74
	4b	0	4
	4c	0	0
Recurrence	with	34	51
	without	75	81

### Immune-Related Genes

The list of 2498 immune-related genes was extracted from the Immunology Database and Analysis Portal (ImmPort) database (https://immport.niaid.nih.gov) (14).

### Development and Verification of an Immune-Related Prognostic Signature

Using univariate Cox regression analysis, the correlation between immune-related genes and prognosis was determined in the training set. Genes with p-value<0.001 were screened as prognostic factors. Then, least absolute shrinkage and selection operator (LASSO) regression analysis was presented to obtain the optimal candidate genes utilizing the glmnet package (15). In the training set, multivariate cox hazard regression modeling was constructed. The stepwise method based on AIC was utilized for further variable selection. The risk score was calculated on the basis of the coefficient and expression value of each candidate in the training and validation sets.

### Evaluation of the Effectiveness of the Signature in Predicting Recurrence

Time‐dependent receiver operating characteristic (ROC) curve of 1-, 3-, and 5-year was built for the signature *via* the survivalROC package in the two datasets. The area under the curve (AUC) was calculated to evaluate its predictive effectiveness. The optimal cutoff value was determined, which was utilized to divide high- and low- risk groups. Kaplan‐Meier curves of overall survival (OS) and recurrence‐free survival (RFS) were conducted between the two groups *via* the survival package. By log‐rank test, the difference in OS and RFS was compared between the two groups. Univariate and multivariate cox regression analysis was also presented to evaluate the predictive independency of this signature in predicting laryngeal cancer outcome.

### Immune Infiltration

The Cell type identification by estimating relative subsets of RNA transcripts (CIBERSORT) algorithm (http://cibersort.stanford.edu/) was utilized to evaluate the proportion of 22 kinds of immune cells in each laryngeal cancer samples from TCGA database (16). A total of 1,000 simulations were presented, and p < 0.05 was significant.

### Construction of a Prognostic Nomogram

Genes in the prognostic model were incorporated into a nomogram for predicting 1-, 3- and 5-year survival probabilities using the rms package.

### Drug Sensitivity Evaluation

The sensitivity to chemotherapy drugs was curated from the Genomics of Drug Sensitivity in Cancer (GDSC; https://www.cancerrxgene.org/) project [15]. The half maximal inhibitory concentration (IC50) was determined with ride regression analysis utilizing the pRRophetic package.

### Patients and Specimens

Postoperative tissue specimens of 26 patients with laryngeal cancer from the Department of Otolaryngology Head and Neck Surgery of the Shengjing Hospital of China Medical University were selected between 2019 and 2020. At the same time, corresponding adjacent tissues (>2 cm from the tumor margin) were selected as controls. All the selected tissue specimens were diagnosed as laryngeal squamous cell carcinoma by the Department of Pathology. All patients provided written informed consent. This research obtained the approval by the Ethics Committee of Shengjing Hospital of China Medical University (2019030).

### Western Blot

Tissues or cells were added with RIPA lysate to extract total cell protein. Protein concentration was determined by BCA method. The 30 μg protein sample was mixed with the loading buffer thoroughly. The protein was isolated by sodium dodecyl sulfate - polyacrylamide gel electrophoresis (SDS-PAGE). After the reaction, the membrane was transferred and sealed, and the primary protein antibody including RCN1 (1/1000; ab205927; Abcam, USA), DNAJA2 (1/10000; ab168365; Abcam, USA), LASP1 (1/10000; ab156872, USA), IBSP (1/1000; A-AP14114a; Abgent, USA), β-actin (1/5000; ab179467; Abcam, USA), LAT2 (1/1000; ab75610; Abcam, USA), FUZ (1/1000; ab111842; Abcam, USA), HOOK2 (1/1000; ab133691; Abcam, USA), and DAPK2 (1/1000; ab111928; Abcam, USA) was added and incubated at 4°C for 24 h. After TBST washing, IgG secondary antibody (1/5000; ab7090; Abcam, USA) was added. ECL was added, and the protein bands were analyzed by automatic gel imaging system.

### Real-Time Quantitative Polymerase Chain Reaction (RT-qPCR)

Total RNA was extracted from laryngeal cancer and paracancerous tissues by Trizol method. The quality of RNA was measured by ultraviolet spectrophotometer, and cDNA was synthesized by reverse transcription kit. The primers of RCN1 (5’-AAACGGGTGCAGAAAAGATACA-3’ (forward) and 5’-AGGTAGTAACCATAGGTGGCTT-3’ (reverse)); DNAJA2 (5’-GTGGCTGACACGAAGCTGTA-3’ (forward) and 5’-AAGACCTTGCTCTCCGTATCT-3’ (reverse)); LASP1 (5’-TGCGGCAAGATCGTGTATCC-3’ (forward) and 5’-GCAGTAGGGCTTCTTCTCGTAG-3’ (reverse)); IBSP (5’-CACTGGAGCCAATGCAGAAGA-3’ (forward) and 5’-TGGTGGGGTTGTAGGTTCAAA-3’ (reverse)); LAT2 (5’-ACAGAGCTTTACGGGGTCC-3’ (forward) and 5’-TGGGGTCTATGTAGGCTTCCT-3’ (reverse)); FUZ (5’-GACTTGAGGGCCAGTTATTGC-3’ (forward) and 5’-GACACCACCAGACTGACGA-3’ (reverse)); HOOK2 (5’- AAGCTGAGCTATGCGGGTC-3’ (forward) and 5’-AGGAGGGGTCTATCTGGTTCA-3’ (reverse)); DAPK2 (5’- CATCCTTGAGCTAGTGTCTGGA-3’ (forward) and 5’-GGATCTGCTTAATGAAGCTGGT-3’ (reverse)) and β-actin (5’-TGCTGTCCCTGTATGCCTC-3’ (forward) and 5’-TGATGTCACGCACGCAGATTT-3’ (reverse)) were synthesized by Shanghai Bioengineering Co., Ltd. (China). The reaction system was prepared according to the instructions of the qRT-PCR detection kit. The system was presented in an ABI 7500 RT-qPCR instrument. The reaction conditions included: pre-denaturation at 95°C for 5 min, denaturation at 95°C for 15 s, annealing at 60°C for 60 s, and extension at 72°C for 30 s, with a total of 40 cycles. β-actin served as an internal control of RCN1. By applying the 2^-ΔΔCt^ method, RCN1 mRNA expression was quantified.

### Cell Culture and Transfection

TU686 and TU212 cells (ATCC, USA) were cultured in DMEM medium (Shanghai Yubo Biotechnology Co., Ltd, China) containing 10% FBS and placed in an incubator with 5% CO_2_ at 37°C. The cells were sub-cultured when the growth density reached about 85%. The cells in logarithmic growth phase were inoculated in a 6-well plate and transfected when the cell fusion reached 70%. The operation was carried out according to the instructions of Lipofectamine2000 reagent (Invitrogen, USA). The culture medium was changed to the medium without FBS 1 h before transfection. The siRNA negative control (si-NC) or siRNA against RCN1 (siRCN1; sequence: 5’-GGAUGAGAAGCUAACUAAAGA-3’) was transfected into the cells. The medium containing FBS was replaced after 6 h transfection and continued for 48 h. The cells were collected for subsequent experiments.

### Transwell

The upper chamber at the bottom of the transwell chamber was added with Matrigel gel (100 μL/well). Transfected TU686 and TU212 cells were collected and resuspended (2×10^5/^mL). The resuspended cells were added to the upper chamber of the transwell chamber (200 μl/well). 600 μl DMEM medium containing FBS was added to the lower chamber of transwell. The cells were placed in an incubator with 5% CO_2_ at 37°C for 24 h. The cells were fixed with paraformaldehyde for 20 min. Then, the cells were washed with PBS. They were stained with crystal violet solution for 15 min. After washing with PBS, the number of invaded cells was observed under the microscope.

### Wound Healing

The transfected cells were collected and counted after trypsin digestion. Then, the cells were seeded into 6-well plates (1×10^4^/mL). 24 h after inoculation, the 6-well plate was scratched, and washed with PBS for 3 times. The serum-free medium was replaced. The migration of cells was observed at 0 h and 24 h after scratches.

### Statistical Analyses

All data were analyzed using R language (version: 3.6.1; http://www.r-project.org/). Comparisons between groups were presented with student’s t test or Wilcoxon test. Pearson or Spearmon correlation test was presented for evaluating the association between variables. Statistical significance was set as p < 0.05.

## Results

### Patient Characteristics

RNA-seq data of 51 recurrence laryngeal cancer patients and 81 non-recurrence patients were from TCGA database, as the training set (**Table 1**). Microarray expression profiles of 34 recurrence laryngeal cancer patients and 75 non-recurrence patients were selected as the validation set. In the training set, 62 patients were aged ≤60. For grade, patients were diagnosed at 1 (n = 6), 2 (n = 18), 3 (n = 30), 4a (n = 74) and 4b (n = 4). In the validation set, 43 patients were aged ≤60. For grade, all patients were diagnosed at 1 (n = 44), 2 (n = 49) and 3 (n = 16).

### Development of an Immune Signature for Predicting Laryngeal Cancer Recurrence

**Figure 1:**
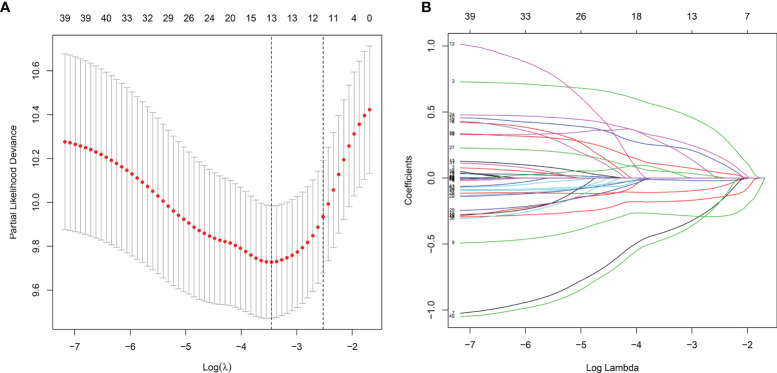
The LASSO coefficient profiles of immune-related genes. The optimal number of variables was determined using LASSO regression analysis. **(A)** 10-fold cross verification for selecting lambda in the LASSO model on the basis of the minimum criteria. **(B)** The optimal number of variables was determined using LASSO regression analysis.

Totally, 2498 immune genes were extracted from the IMMPORT database. In the training set, prognosis-related genes with p-value <0.001 were screened by single factor cox regression. A total of 43 immune genes were identified as the most significant factors affecting the prognosis. When the number of variables was 13, the partial likelihood deviation was the smallest (**Figure 1A**). The regression coefficients of the 13 variables were calculated in the LASSO model (**Figure 1B**). Multivariate cox hazard regression analysis was then carried out in the training set, and the stepwise method based on AIC was used for further variable selection. Finally, an eight immune-related signature was conducted, composed of RCN1, LAT2, DAPK2, DNAJA2, FUZ, LASP1, IBSP and HOOK2 (**Table 2**). Among them, RCN1 (hazard ratio (HR): 1.928852, 95% confidence interval (CI): 1.194018-3.115926, p-value = 0.007261) and IBSP (HR: 1.928852, 95% CI: 1.194018-3.115926, p-value = 0.007261) were risk factors for laryngeal cancer. Moreover, LAT2 (HR: 0.525901, 95% CI: 0.320208-0.863728, p-value = 0.011127) and HOOK2 (HR: 0.458976, 95% CI: 0.25448-0.827801, p-value = 0.009654) were protective factors for laryngeal cancer. The risk score of each sample was calculated, as follows: risk score = 0.656925 * the expression level of RCN1 + (-0.64264) * the expression level of LAT2 + (-0.26934) * the expression level of DAPK2 + 0.380614 * the expression level of DNAJA2 + (-0.28219) * the expression level of FUZ + (0.709185) * the expression level of LASP1 + 0.412168 * the expression level of IBSP + (-0.77876) * the expression level of HOOK2.

**Table 2:** Prognostic characteristics of eight genes in the signature.

ID	coef	HR	95%CI	p-value
RCN1	0.656925	1.928852	1.194018 - 3.115926	0.007261
LAT2	-0.64264	0.525901	0.320208 - 0.863728	0.011127
DAPK2	-0.26934	0.763886	0.532696 - 1.095412	0.143068
DNAJA2	0.380614	1.463182	0.892372 - 2.399116	0.131397
FUZ	-0.28219	0.754132	0.540352 - 1.052491	0.097083
LASP1	0.709185	2.032335	0.882129 - 4.682292	0.095826
IBSP	0.412168	1.510088	1.163374 - 1.960132	0.001955
HOOK2	-0.77876	0.458976	0.25448 - 0.827801	0.009654

coef, coefficients; HR, hazard ratio; CI, confidence interval.

### Evaluation of the Predictive Efficacy of the Signature for Laryngeal Cancer Recurrence

**Figure 2:**
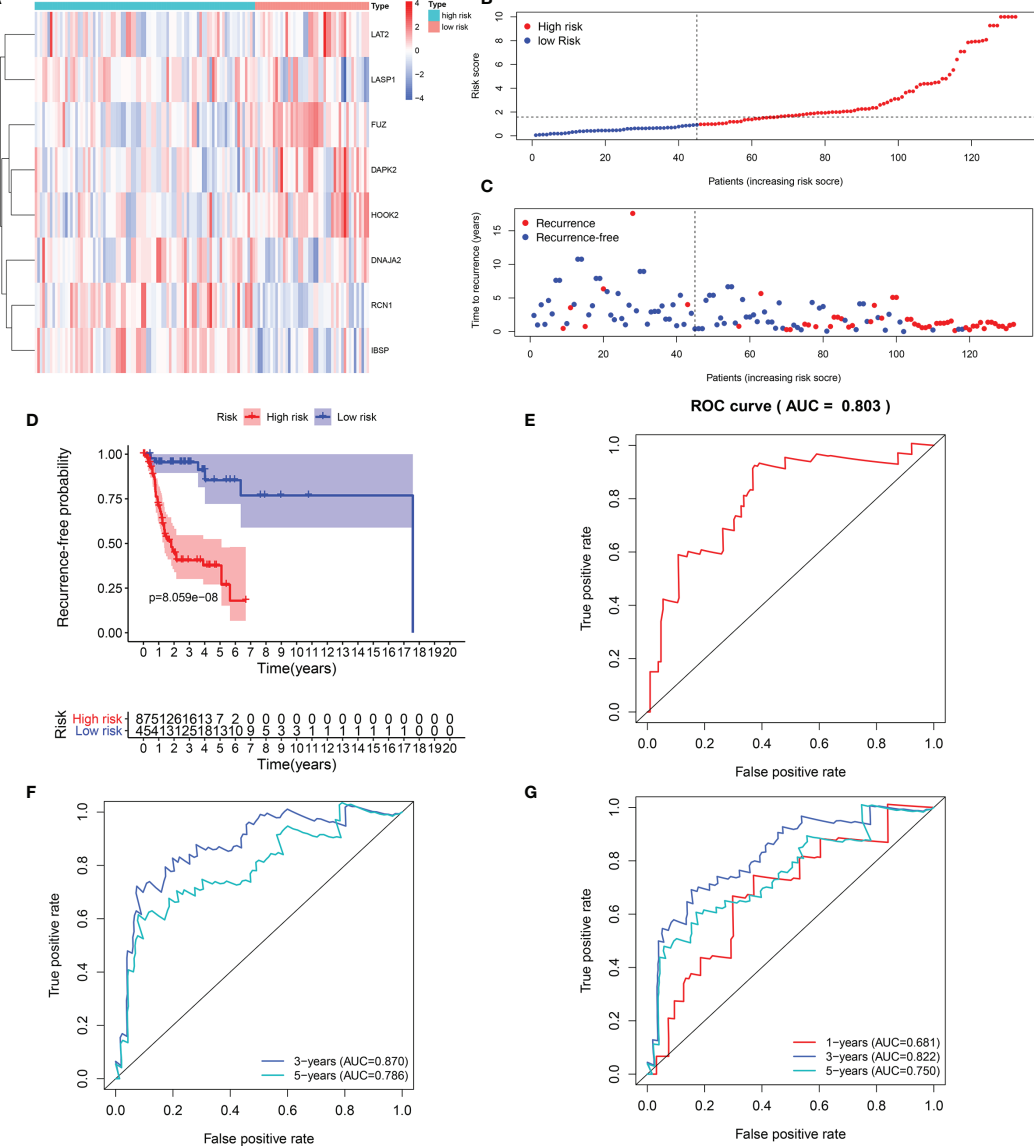
Evaluation of the predictive efficacy of the signature for laryngeal cancer recurrence in the training set. **(A)** Heat maps depicting the expression patterns of these eight genes including RCN1, LAT2, DAPK2, DNAJA2, FUZ, LASP1, IBSP and HOOK2 between high- and low- risk score group. Red: up-regulation and blue: down-regulation. **(B)** Ranking of risk scores among all laryngeal cancer patients. **(C)** Distribution of recurrence time among all laryngeal cancer patients. Red dots express recurrence laryngeal cancer samples and blue dots represent non-recurrence samples. The dotted line indicates the optimal cutoff value of risk score. The left side of the line represents low-risk patients, while the right side represents high-risk patients. **(D)** Recurrence-free survival between high- and low- risk score groups. **(E)** ROC curve for predicting the patients’ 1-year recurrence. **(F)** ROC curves for predicting the patients’ 3 and 5-year recurrence. **(G)** ROC curves for prediction of 1, 3- and 5-year OS outcome.

Heat maps visualized the differences in expression patterns of these eight genes between high-and low- risk score groups in the training set (**Figure 2A**). We determined the optimal cutoff value to divide the high and low risk groups, and the optimal cutoff was 1.681355 (**Figure 2B**). As the risk score of patients with laryngeal cancer increased, the number of red dots gradually increased, indicating an increase in the number of patients with recurrence (**Figure 2C**). Thus, the high-risk population had a higher recurrence rate. Kaplan-Meier RFS results demonstrated that patients with high risk score indicated a higher recurrence risk than those with low risk score (p=8.059-08; **Figure 2D**). The ROC curve of the model was drawn to evaluate its sensitivity and specificity. In **Figure 2E**, the AUC of the constructed immune model predicting the patients’ 1-year recurrence rate was 0.803. Additionally, the AUC values at 3- and 5-year recurrence were separately 0.870 and 0.786 (**Figure 2F**). This indicated that the model exhibited the well efficacy for the prediction of recurrence. We also conducted the ROC curves at 1-, 3- and 5-year OS outcome and our results showed that the AUC values at 1, 3- and 5-year were separately 0.681, 0.822 and 0.750 (**Figure 2G**), demonstrating that this signature could also predict patients’ OS outcome.

### Validation of the Predictive Efficacy of the Signature for Predicting Recurrence of Laryngeal Cancer

**Figure 3:**
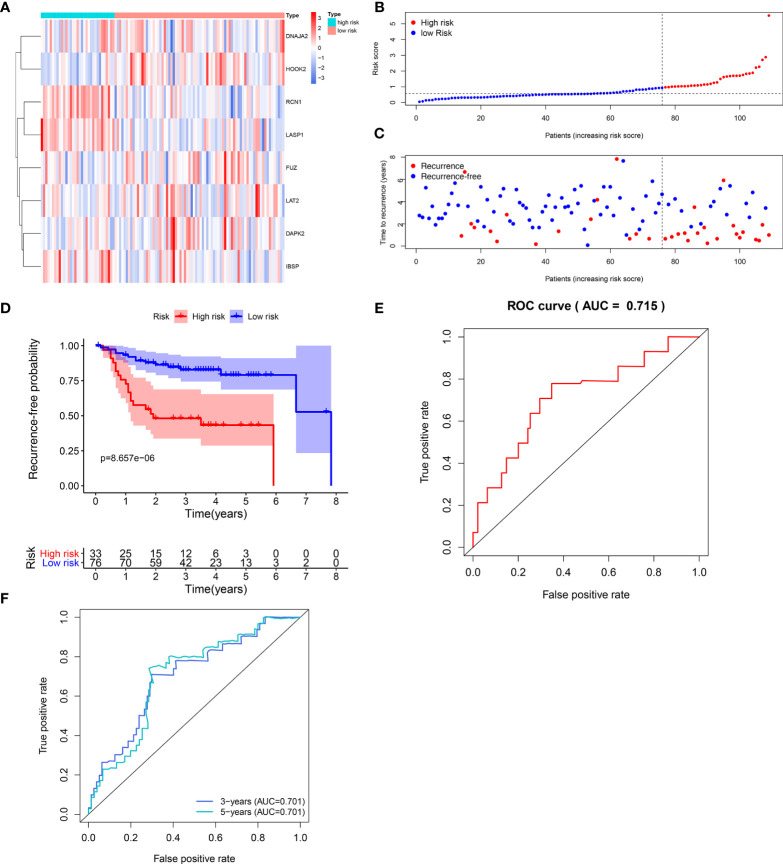
Validation of the predictive efficacy of the signature for predicting recurrence of laryngeal cancer in the verification set. **(A)** Heat maps visualizing the differences expression patterns of these eight genes including RCN1, LAT2, DAPK2, DNAJA2, FUZ, LASP1, IBSP and HOOK2 between high- and low- risk score group. Red expresses up-regulation and blue indicates down-regulation. **(B)** Ranking of risk scores among all laryngeal cancer patients. **(C)** Distribution of recurrence time among all laryngeal cancer patients. Red dots express recurrence laryngeal cancer samples and blue dots represent non-recurrence samples. The dotted line indicates the optimal cutoff value of risk score. The left side of the line represents low-risk patients, while the right side represents high-risk patients. **(D)** Recurrence-free survival between high- and low- risk score groups. **(E)** ROC curve for predicting the patients’ 1-year recurrence. **(F)** ROC curves for predicting the patients’ 3 and 5-year recurrence.

The efficacy of predicting recurrence of the signature was verified in the validation set. As depicted in the heat maps, we visualized the expression patterns of these eight genes between high-and low- risk score groups in the validation set (**Figure 3A**). There were distinct differences in their expression between the two groups. Following determination of the optimal cutoff value, 109 laryngeal cancer samples were separated into high- and low- risk score groups (**Figure 3B**). As the risk score of patients with laryngeal cancer increased, the number of samples with recurrence gradually increased (**Figure 3C**). Therefore, high-risk score patients exhibited a distinctly higher recurrence risk. Our Kaplan-Meier RFS results suggested that patients with high risk score usually experienced a higher recurrence risk in comparison to those with low risk score (p=8.657-06; **Figure 3D**). The AUC values of the ROC curve for patients’ 1- (**Figure 3E**), 3- and 5-year recurrence (**Figure 3F**) were 0.715, 0.701 and 0.701, indicating that the signature had the well efficacy for predicting recurrence risk of laryngeal cancer. Additionally, our uni- and multivariate cox regression analysis results demonstrated that the prognostic model was a clinically independent prognostic factor (**Table 3**).

**Table 3:** Uni- and multivariate cox regression analysis both in the training and validations sets.

Cohort	Parameters	Univariable Cox regression		Multi-variable Cox regression	
		HR (95%CI)	p	HR (95%CI)	p
Training	Age	1.001 (0.969-1.035)	0.940	0.998 (0.964-1.034)	0.928
	Gender	0.638 (0.295-1.380)	0.253	0.678 (0.305-1.505)	0.339
	Grade	1.036 (0.605-1.773)	0.897	1.136 (0.618-2.087)	0.682
	Risk Score	5.206 (3.108-8.720)	P<0.0001	5.170 (3.086-8.661)	P<0.0001
Validation	Age	1.023 (0.989-1.058)	0.192	1.008 (0.974-1.044)	0.640
	Grade	1.037 (0.632-1.702)	0.886	0.846 (0.503-1.423)	0.528
	Risk Score	3.521 (1.749-7.091)	0.000	3.443 (1.665-7.117)	0.001

### Characteristics of Immune Infiltration of the Signature

**Figure 4:**
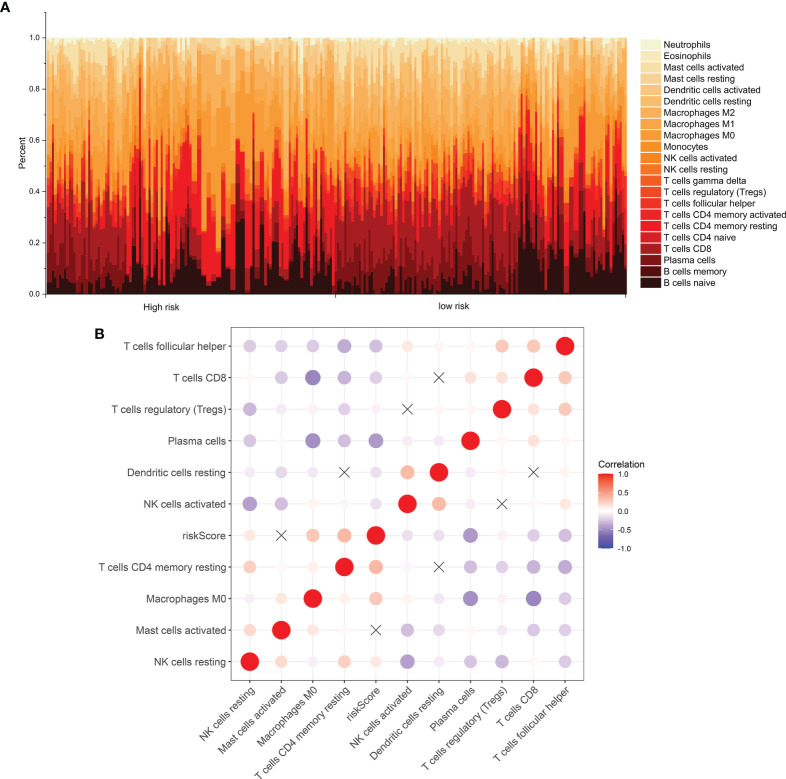
Immune cell infiltration analysis. **(A)** The distribution of 22 kinds of immune cells between high- and low- risk groups *via* the CIBERSORT algorithm. **(B)** Heatmap showing the interaction between immune cells and the risk score among laryngeal cancer patients.

**Figure 5:**
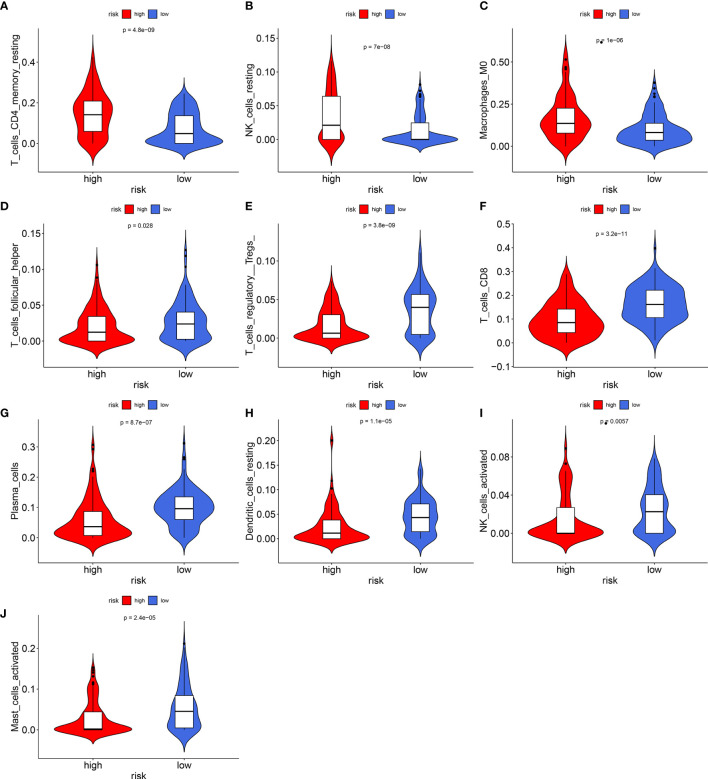
Violin plots showing the relationship between the signature and infiltration levels of 22 kinds of immune cells. **(A)** T cells CD4 memory resting; **(B)** NK cells; **(C)** macrophage M0; **(D)** T cells follicular helper; **(E)** T cells regulatory; **(F)** T cells; **(G)** plasma cells; **(H)** dendritic cells; **(I)** NK cells; **(J)** mast cells activated. Red indicates high recurrence risk group and blue indicates low recurrence risk group.

The levels of immune infiltration of 22 kinds of immune cells were assessed between high recurrence risk and low recurrence risk groups using the CIBERSORT algorithm (**Figure 4A**). In **Figure 4B**, we investigated that the signature was positively correlated to NK cells resting, macrophages M0 and T cells DC4 memory resting but was negatively correlated to NK cells activated, dendritic cells resting, plasma cells, Tregs, T cells CD8 and T cells follicular helper. There were higher infiltration levels of T cells CD4 memory resting (p=4.8e-09; **Figure 5A**), NK cells (p=7e-08; **Figure 5B**), macrophage M0 (p=1e-06; **Figure 5C**) in samples with high recurrence risk in comparison to those with low recurrence risk. Moreover, samples with high recurrence risk exhibited lower infiltration levels of T cells follicular helper (p=0.028; **Figure 5D**), T cells regulatory (Tregs; p=3.8e-09; **Figure 5E**), T cells CD8 (p=3.2e-11; **Figure 5F**), plasma cells (p=8.7e-07; **Figure 5G**), dendritic cells (p=1.1e-05; **Figure 5H**), NK cells (p=0.0057; **Figure 5I**) and mast cells activated (p=2.4e-05; **Figure 5J**) compared to those with low risk.

### Evaluation of the Clinical Application Potential of the Prognostic Signature

**Figure 6:**
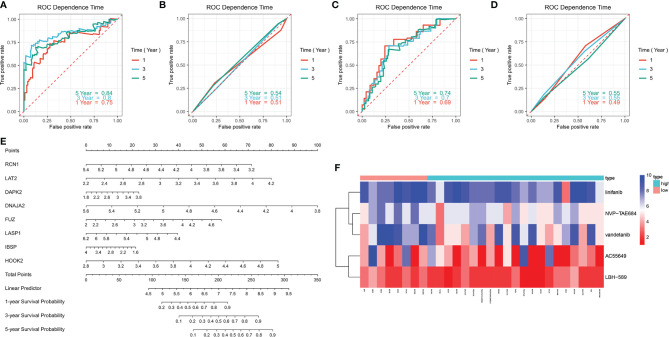
Evaluation of the clinical predictive efficacy of the signature and drug sensitivity. **(A, B)** Comparison of the predictive efficacy between **(A)** the signature and **(B)** grade in the training set in accordance with ROC curves at 1-, 3- and 5-year recurrence. **(C, D)** Comparison of the predictive efficacy between **(C)** the signature and **(D)** grade in the validation set based on ROC curves at 1-, 3- and 5-year recurrence. **(E)** Construction of a prognostic nomogram for predicting patients’ 1-, 3- and 5-year recurrence. **(F)** Heatmap showing the difference in drug sensitivity between high- and low-risk groups.

ROC curves were conducted to compare the predictive performance of the prognostic signature with that of grade. In the training set, higher AUC values at 1-, 3- and 5-year recurrence were investigated in the prognostic model (**Figure 6A**) in compared with grade (**Figure 6B**). The similar results were found in the validation set (**Figures 6C, D** **)**. This indicated that the prognostic signature possessed the better predictive performance than grade. For facilitating the clinical practice, we established a prognostic nomogram for predicting 1-, 3-, and 5-year survival probabilities based on the genes in the prognostic signature (**Figure 6E**).

### Comparison of the Effects for Patients in High- and Low Risk to Receive Chemotherapy

We also compared the effects for patients in high- and low risk to receive chemotherapy. Our results demonstrated that high-risk group presented significantly lower IC50 values of NVP-TAE684 (p=0.013111), vandetanib (p=0.015979), AC55649 (p=0.025122), LBH-589 (p=0.042172) and linifanib (p=0.045183) compared with low-risk group (**Table 4** **;** **Figure 6F**). This indicated that high-risk patients were more sensitive to above chemotherapeutic drugs.

**Table 4:** Comparison of the drug sensitivity between high- and low-risk groups.

Drugs	Mean IC50 (μM)		p
	High-risk	Low-risk	
NVP-TAE684	2.724381	4.350444	0.013111
vandetanib	4.079224	16.53434	0.015979
AC55649	1.432637	1.1E+08	0.025122
LBH-589	0.060236	0.135834	0.042172
linifanib	7.86301	13.45067	0.045183

### Validation of the Genes in the Prognostic Signature

**Figure 7:**
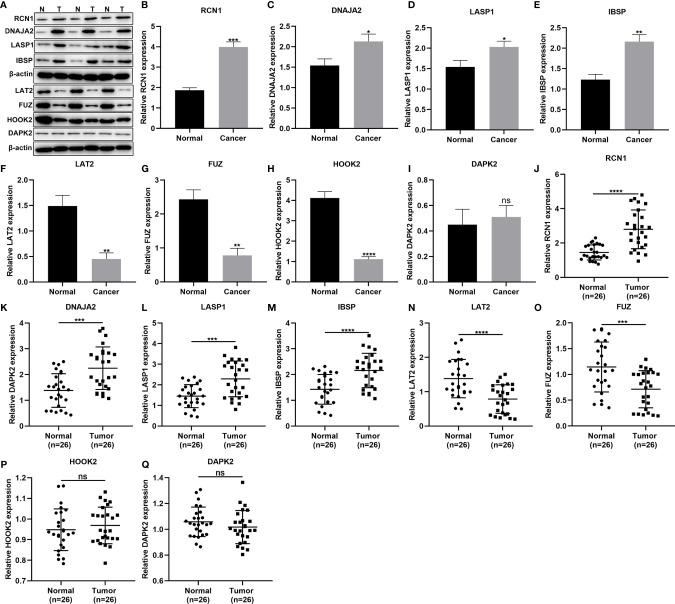
Validation of the genes in the prognostic signature. **(A–I)** Western blot for detection of the protein expression of **(B)** RCN1, **(C)** DNAJA2, **(D)** LASP1, **(E)** IBSP, **(F)** LAT2, **(G)** FUZ, **(H)** HOOK2, and **(I)** DAPK2 in laryngeal cancer and normal tissues. **(J–Q)** RT-qPCR for detecting the mRNA expression of **(J)** RCN1, **(K)** DNAJA2, **(L)** LASP1, **(M)** IBSP, **(N)** LAT2, **(O)** FUZ, **(P)** HOOK2, and **(Q)** DAPK2 in laryngeal cancer and normal tissues. Ns, not significant; *P < 0.05; **p < 0.01; ***p < 0.001; ****p < 0.0001.

This study included 26 laryngeal cancer patients in our study. **Table 5** shows the clinical features of above patients. The genes in the signature were detected in three paired laryngeal cancer and normal tissues by western blot (**Figure 7A**). As a result, RCN1 (p < 0.001; **Figure 7B**), DNAJA2 (p < 0.05; **Figure 7C**), LASP1 (p < 0.05; **Figure 7D**) and IBSP (p < 0.01; **Figure 7E**) exhibited higher expression while LAT2 (p < 0.01; **Figure 7F**), FUZ (p < 0.01; **Figure 7G**), and HOOK2 (p < 0.0001; **Figure 7H**) presented lower expression in laryngeal cancer than normal tissues. However, no significance difference in DAPK2 expression was found in laryngeal cancer and normal tissues (**Figure 7I**). We also detected the mRNA expression of above genes in 26 paired laryngeal cancer and normal tissues *via* RT-qPCR. As a result, RCN1 (p < 0.0001; **Figure 7J**), DNAJA2 (p < 0.001; **Figure 7K**), LASP1 (p < 0.001; **Figure 7L**) and IBSP (p < 0.0001; **Figure 7M**) were markedly up-regulated while LAT2 (p < 0.0001; **Figure 7N**) and FUZ (p < 0.0001; **Figure 7O**) were remarkably down-regulated in laryngeal cancer compared with normal tissues. Nevertheless, there was no significant difference in HOOK2 (**Figure 7P**) and DAPK2 (**Figure 7Q**) between laryngeal cancer and normal tissues.

**Table 5:** Clinicopathological parameters of 26 laryngeal cancer patients in our cohort.

Clinicopathological parameters	N
Gender	
male	24
female	2
Age, years	
<60	11
≥60	15
Tobacco	
NO	5
YES	21
T status	
T1-T2	14
T3-T4	12
Lymph node metastasis	
No	19
Yes	7
TNM clinical stage	
I-II	12
III-IV	14
Pathology grade	
Well/moderate	15
Poor/undifferentiated	11
Tumor location	
Glottic	16
Supraglottic	10

### Knockdown of RCN1 Lowers Invasion of Laryngeal Cancer Cells

**Figure 8:**
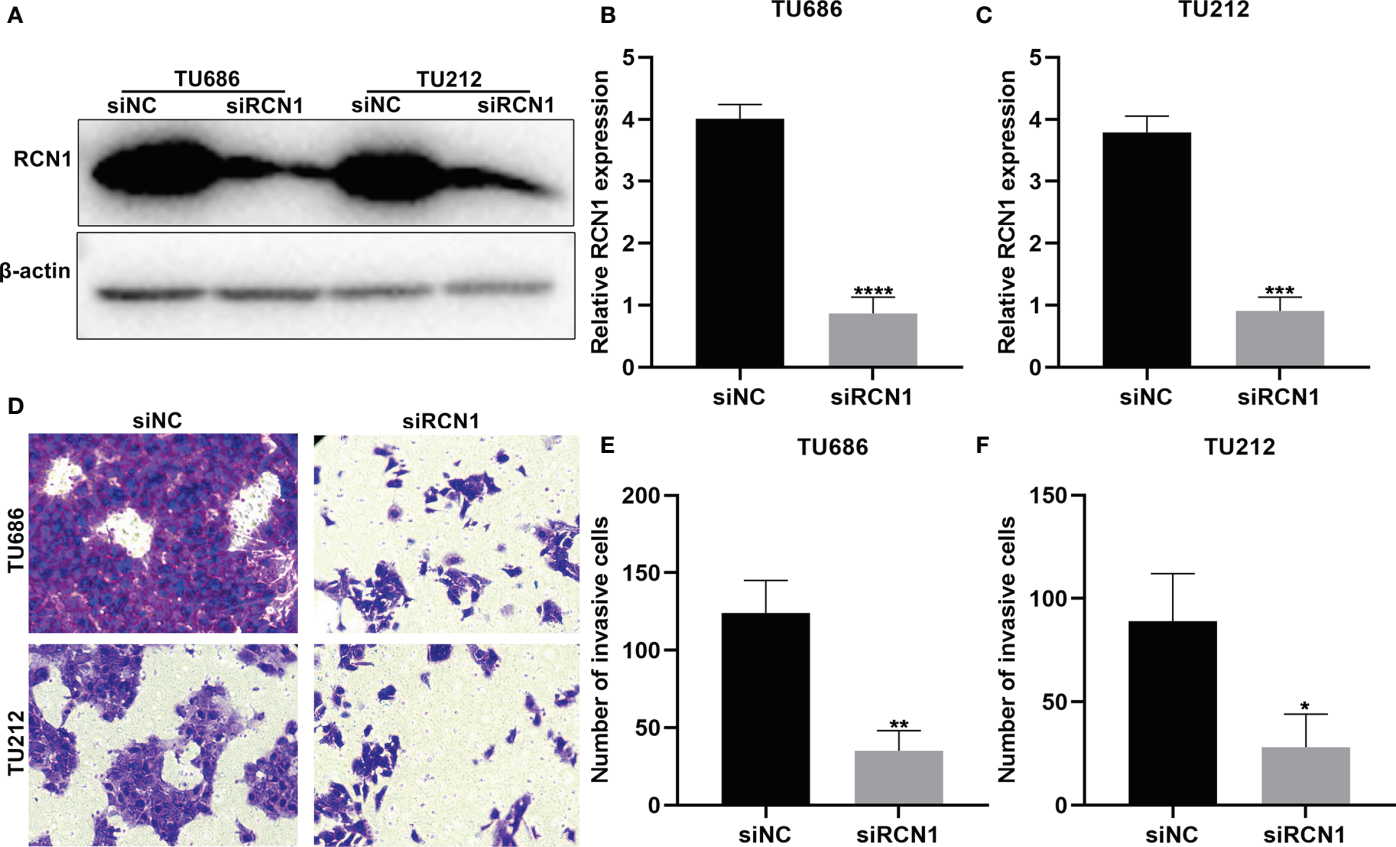
RCNA1 knockdown lowers invasion of laryngeal cancer cells. **(A–C)** Western blot for detection of RCNA1 expression in TU686 and TU212 cells transfected with siRCNA1. **(D–F)** Transwell for detection of the invasion of TU686 and TU212 cells transfected with siRCNA1. *P < 0.05; **p < 0.01; ***p < 0.001; ****p < 0.0001.

To investigate the functions of RCN1 in laryngeal cancer progression, siRCN1 was transfected into TU686 and TU212 cells. Western blot confirmed that RCN1 expression was distinctly decreased in TU686 (p < 0.0001) and TU212 cells (p < 0.001; **Figures 8A–C**). After silencing RCN1, invasion of laryngeal cancer cells was assessed through transwell assay. Our data demonstrated that the invasive capacity of TU686 (p < 0.01) and TU212 (p < 0.01) cells was significantly restrained by siRCN1 transfection (**Figures 8D–F**).

### Knockdown of RCN1 Restrains Migration of Laryngeal Cancer Cells

**Figure 9:**
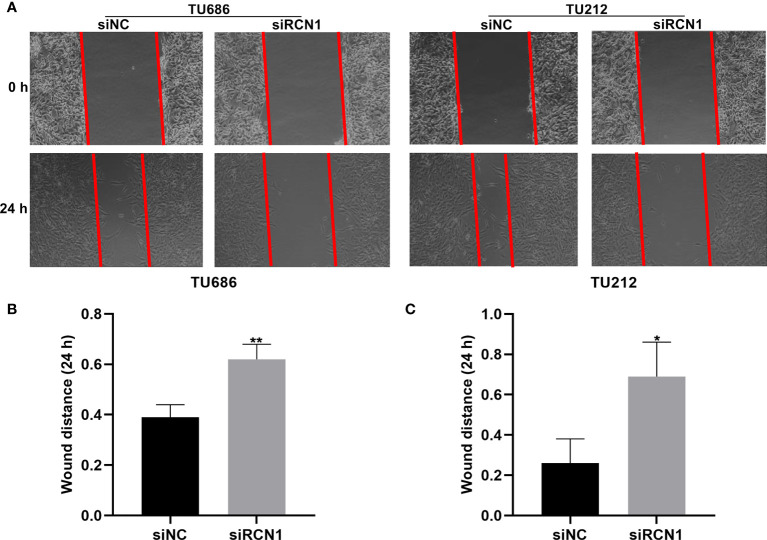
RCNA1 knockdown restrains migration of laryngeal cancer cells. **(A)** Representative images of wound healing assay. **(B, C)** Quantification of wound distance of TU686 and TU212 cells transfected with siRCNA1. *P < 0.05; **p < 0.01.

The migrated ability of laryngeal cancer cells was evaluated by wound healing. As a result, wider wound distance was found in TU686 (p < 0.01) and TU212 (p < 0.05) cells under transfection with siRCN1 (**Figures 9A–C**). The data demonstrated that silencing RCN1 may restrain migration of laryngeal cancer cells.

## Discussion

Recurrence is the most common cause of death in postoperative patients with laryngeal cancer. There are no reliable clinical tools to identify patients who may benefit from adjuvant therapy (17). Our findings developed and verified an immune-related signature for prediction of laryngeal cancer recurrence. Patients with high risk score had higher risk of recurrence. Thus, these patients should take more radical treatments. Our ROC curve confirmed its well performance for predicting laryngeal cancer recurrence.

Increasing markers related to laryngeal cancer recurrence may be found in recent studies (18). Prognostic guidance has been implemented to determine whether organ is preserved. In patients with recurrent laryngeal cancer, clinical outcome is still not the optimal choice. Most of them may not be suitable for surgery. Due to the importance of immune infiltration in laryngeal cancer recurrence, the newly approved immunotherapy could bring distinct benefit to these patients (18). Patients with recurrence may be the main candidates for immune adjuvant therapy. How to identify this subgroup of patients still faces challenges. In this study, we developed an immune-related signature for predicting laryngeal cancer recurrence. This signature was composed of RCN1, LAT2, DAPK2, DNAJA2, FUZ, LASP1, IBSP and HOOK2. Our data confirmed that RCN1, DNAJA2, LASP1 and IBSP were up-regulated while LAT2, FUZ, HOOK2 and DAPK2 were down-regulated in laryngeal cancer. RCN1 knockdown restrained migrated and invasive abilities of laryngeal cancer cells. Dysregulated RCN1 is found in multiple kinds of cancers including non-small cell lung cancer (NSCLC) (19), prostate cancer (20), renal cell carcinoma (21), nasopharyngeal carcinoma (22), and oral squamous cell carcinoma (23). Its knockdown accelerates tumor cell apoptosis and necrosis (20). Nevertheless, RCN1 expression is still undiscovered in laryngeal cancer. Our results firstly demonstrated that RCN1 was a risk factor for laryngeal cancer. For patients in the high-risk recurrence group, its expression was higher than those in the low-risk recurrence group following validation. The roles of LAT2 have been widely studied in different cancers (24). For example, LAT2 as an oncogene could weaken the sensitivity of pancreatic cancer cells to gemcitabine (25). Consistent with other cancers, its expression is up-regulated in laryngeal cancer patients with high risk recurrence (26). Down-regulated DAPK1 has a relationship to tumor recurrence as well as metastasis (27). DAPK is frequently hypermethylated in laryngeal cancer tissues than controls (28). Hypermethylation of tumor suppressor genes induced by risk factors such as smoking and drinking contribute to progression of laryngeal cancer (29). Herein, we found that DAPK was often lowly expressed in laryngeal cancer patients with high risk recurrence, indicating that the loss of its expression might accelerate recurrence of laryngeal cancer. FUZ promotes the progression and metastasis of NSCLC (30). High FUZ expression implies poor prognosis for NSCLC patients. However, no study has explored the function of FUZ in laryngeal cancer. LASP1 is in relation to a variety of human malignancies (31). It is highly expressed in laryngeal cancer tissues, which promotes the invasion and proliferation of laryngeal cancer cells (32). In this study, patients with high recurrence risk had higher LASP1 expression than those with low risk. Combining previous studies, overexpressed LASP1 could be involved in recurrence of laryngeal cancer. The roles of IBSP in head and neck tumors have been studied. For instance, IBSP is highly expressed in esophageal squamous cell carcinoma (ESCC) tissues (33). Up-regulation of IBSP is positively correlated with lymph node metastasis, TNM stage as well as poor clinical outcomes for ESCC patients (33). Furthermore, it is associated with metastasis and recurrence of ESCC (33) and breast cancer (34). Our research demonstrated that it was highly expressed in laryngeal cancer tissues with high recurrence risk in comparison to low risk. Moreover, it was a risk factor for laryngeal cancer recurrence. HOOK2 is detected in serum of ESCC patients (35). Its low expression was found in laryngeal cancer with high recurrence risk. Also, it was a protective factor for laryngeal cancer recurrence. The molecular mechanisms involving these eight genes remain undiscovered in laryngeal cancer recurrence.

We assessed the immune infiltration levels of 22 kinds of immune cells between high- and low recurrence risk groups using the CIBERSORT algorithm. Laryngeal cancer samples with high recurrence risk exhibited higher infiltration levels of T cells CD4 memory resting, NK cells, macrophage M0 in comparison to those with low recurrence risk. Samples with high recurrence risk exhibited lower infiltration levels of T cells follicular helper, Tregs, T cells CD8, plasma cells, dendritic cells, NK cells and mast cells activated compared to those with low risk. Immune cells in the tumor microenvironment have been confirmed to be related to laryngeal cancer recurrence (18). For example, CD4 and CD8 tumor-infiltrating lymphocytes show the potential to predict the prognosis of patients with recurrent laryngeal cancer (36). Tumor-associated macrophage is an independent factor for prediction of the overall survival of patients with laryngeal cancer (37). Thus, interaction between this signature and immune cells could contribute to recurrence of laryngeal cancer.

The innovations of this research are as follows: (1) Recurrence remains a major obstacle to long-term survival of postoperative patients with laryngeal cancer. Nevertheless, limited genetic models can precisely predict recurrence probability and optimize therapeutic strategies for laryngeal cancer. The immune-related signature we proposed can provide accurate estimations of the recurrence prediction as well as might offer novel research directions and prospects for individualized treatment of patients with laryngeal cancer. (2) This study collected fresh clinical specimens to confirm the application of our model from biological algorithm approaches. (3) The biological function of one gene in the model was confirmed through *in vitro* experiments. Although our results are encouraging, several limitations should be pointed out. This study was based on the two public databases. The number of laryngeal cancer patients was relatively limited. Furthermore, this was a retrospective study. The immune-related recurrence signature should be verified in a prospective study.

## Conclusion

Taken together, we developed and verified an immune-related signature for predicting the recurrence of laryngeal cancer. Patients with high risk score had a higher recurrence risk in comparison to those with low risk score. This signature could be correlated to immune infiltration. More clinical studies will be carried out to validate the performance of this signature for prediction of laryngeal cancer recurrence.

## Data Availability Statement

The original contributions presented in the study are included in the article/supplementary material. Further inquiries can be directed to the corresponding author.

## Ethics Statement

This research obtained the approval by the Ethics Committee of Shengjing Hospital of China Medical University (2019030). The patients/participants provided their written informed consent to participate in this study.

## Author Contributions

WJ conceived and designed the study. HZ conducted most of the experiments and data analysis, and wrote the manuscript. XZ and JW participated in collecting data and helped to draft the manuscript. All authors reviewed and approved the manuscript.

## Funding

This work was funded by National Natural Science Foundation of China (81202126, 81072196) and 345 Talent Project (M0710).

## Conflict of Interest

The authors declare that the research was conducted in the absence of any commercial or financial relationships that could be construed as a potential conflict of interest.

## Publisher’s Note

All claims expressed in this article are solely those of the authors and do not necessarily represent those of their affiliated organizations, or those of the publisher, the editors and the reviewers. Any product that may be evaluated in this article, or claim that may be made by its manufacturer, is not guaranteed or endorsed by the publisher.

## Abbreviations

TCGA, The Cancer Genome Atlas; GEO, Gene Expression Omnibus; RFS, recurrence free survival; ROC, receiver operating characteristic; AUC, area under curve; TNM, tumor, node, metastasis; ImmPort, Immunology Database and Analysis Portal; LASSO, a least absolute shrinkage and selection operator; CIBERSORT, Cell type identification by estimating relative subsets of RNA transcripts; coef, coefficients; HR, hazard ratio; CI, confidence interval.
